# Desvenlafaxine

**DOI:** 10.4103/0019-5545.58303

**Published:** 2009

**Authors:** Chittaranjan Andrade

**Affiliations:** Department of Psychopharmacology, National Institute of Mental Health and Neurosciences, Bangalore - 560 029, India

Desvenlafaxine (DV) succinate (Pristiq; Wyeth) is a recent serotonin-norepinephrine reuptake-inhibiting (SNRI) antidepressant drug. DV is O-desmethylvenlafaxine, the principal active metabolite of venlafaxine, formed by the action of CYP 2D6 on the parent drug. In February 2008, DV (50 mg/day) was approved for the treatment of depression by the Food and Drug Administration (FDA) in the USA.[1]

If patients receive venlafaxine, DV is formed in their bodies; so, what is gained if venlafaxine is eliminated from the treatment chain and DV is administered directly? A cynical answer is that DV extends the patent life of venlafaxine. Whereas venlafaxine went out of patent in 2008, the extended-release formulation of the drug will go out of patent in 2010. In India, unless a substantial advantage for DV over venlafaxine is demonstrated, the introduction of DV will be considered to be evergreening; i.e., merely an incremental modification of an existing molecule. Evergreening is not recognized for the grant of patents under the Indian Patents Act as modified by the Parliament in 2005.[2]

Therefore, does DV have any advantages over venlafaxine? At present, it is too early to say. There are both positives and negatives for DV. Positives for DV over venlafaxine are:

1. In clinical trials of patients with major depressive disorder, assessments using a visual analogue scale showed that DV (100-400 mg/day) has a greater efficacy than placebo against painful symptoms associated with depression.[1]
2. In clinical trials of patients with major depressive disorder, assessments using the Sheehan Disability Scale showed that DV (50-400 mg/day) has a greater efficacy than placebo against disability associated with depression.[1]
3. DV has demonstrated efficacy against the vasomotor symptoms (hot flushes) of menopause.[3–5]

It is likely, however, that these properties of DV constitute a class action of all antidepressants and are not unique to DV. More specific advantages with DV over venlfaxine are:

1. In order to maximize tolerance, clinicians usually take 7-10 days or longer to uptitrate the dose of venlafaxine to the target of 150-225 mg/day, at which doses venlafaxine becomes dual-acting. In contrast, DV is started at the target dose of 50 mg/day from the very first day.[1] Should it be considered necessary, an escalation to 100 mg/day can be effected within 4-7 days.
2. CYP 2D6 poor metabolizers constitute about 10% of the population. Such persons would not tolerate standard doses of venlafaxine.[6] Unless clinicians order blood levels or pharmacogenomic tests, they will have no way of knowing whether a patient who develops adverse effects with venlafaxine is a poor metabolizer (in whom lower doses would achieve effective blood levels with fewer adverse effects) or merely does not tolerate standard doses of the drug (necessitating drug withdrawal). Venlafaxine will therefore (unnecessarily) be withdrawn in the former subgroup. In contrast, DV is negligibly metabolized in the liver.[7,8] Therefore, genetic variations in metabolic capacity will not influence the tolerability of the drug or influence adverse effect-related drug withdrawal decisions.
3. Venlafaxine is associated with an up to 10% or greater dose-dependent risk of treatment-emergent hypertension. In contrast, in patients who receive DV (50-400 mg/day), there is only an approximately 1-2% risk of sustained increase in diastolic blood pressure to values >90 mmHg or values >10 mmHg above baseline (as compared with a 0.5% risk in patients receiving placebo).[1]
4. Sexual adverse effects are common with venlafaxine. In contrast, sexual adverse effects are placebo-level with DV 50-100 mg/day, rising to noticeably above placebo level (more in men than in women) only with the 400 mg/day dose.[7,9] A caveat here is that sexual adverse effects with DV were recorded based on spontaneous reports and had not been formally evaluated in the clinical trials available so far, and could therefore be underestimated.
5. Venlafaxine is commonly associated with an unpleasant drug discontinuation reaction because its (terminal) half-life is short - about 5 h. This half-life does not vary as a function of immediate-release vs. extended-release formulation. As a result, the drug is substantially washed out of the body 24 h after a single missed dose. This is why discontinuation symptoms may emerge after even a single missed dose of venlafaxine, and why venlafaxine is best dosed at the same time of day each day. Whereas DV is also associated with a discontinuation syndrome,[1] from a theoretical perspective, because its terminal half-life is longer at around 10 h,[1] a drug discontinuation reaction could be less of a problem as compared with venlafaxine. A caveat here is that, as one molecule of venlafaxine yields one molecule of DV upon metabolism, this putative advantage assumes that venlafaxine and DV have subtle pharmacodynamic differences, and that DV does not compensate pharmacodynamically for the hiatus arising from the washout of venlafaxine. The issue can only be resolved through a head-to-head comparison of discontinuation symptoms between the two drugs; these data have so far not been released.

The above notwithstanding, there are some concerns about DV and some regards in which it has yet to establish itself relative to venlafaxine:

1. Many doses of DV in many trials failed to separate from placebo for the primary outcome measure. This failure was more marked in the flexible-dose (200-400 mg/day) studies and with the 200 mg/day dose in the fixed-dose studies; however, failure at the primary outcome measure was also recorded with the 50 and 100 mg/day doses in certain fixed-dose studies. Clearcut separation for all outcome measures with all doses in all studies, and in both fixed- and flexible-dose designs, emerged only in pooled analyses. However, effect sizes remained low (around 0.30 or less) and onset of separation from placebo was late (2-4 weeks).[10–12]
2. Data exist to suggest that venlafaxine is associated with better treatment outcomes than the selective serotonin reuptake inhibitors (SSRIs), and that venlafaxine may benefit patients who fail SSRI trials. No such studies have so far been conducted with DV.

DV is about 11 times more potent an inhibitor of serotonin reuptake than of norepinephrine reuptake.[13] Just as venlafaxine becomes a dual-acting SNRI drug only at higher doses (e.g., 150 mg/day and above), might it be possible that DV is merely an SSRI at lower doses? If so, this might, at least in part, explain some of the disappointing results with the drug in the clinical trials conducted to date. Such a possibility is supported by the adverse effect profile of the drug in 50-100 mg/day doses: nausea and dizziness, which are serotonergic adverse effects, are noticeably present whereas dysuria and hypertension, which are noradrenergic adverse effects, are negligible or absent.[7]

Here, it may be noted that strongly noradrenergic drugs such as reboxetine and more balanced SNRIs such as duloxetine and milnacipran have both found poor acceptance in clinical practice and/or have evidenced unimpressive results relative to SSRIs in meta-analyses. If DV is more strongly noradrenergic than venlafaxine, this may explain the somewhat unimpressive clinical trial results. However, as noted above, the adverse effect profile of the drug does not seem to suggest a noradrenergic bias.

With this background, mark *True* or *False* against each of the following statements:

1. The dose-dependent risk of dry mouth and constipation with DV is due to muscarinic receptor blockade.
2. Patients prescribed DV may lose weight.
3. DV results in a clinically significant increase in the QTc interval.
4. DV inhibits CYP 2D6.
5. DV should be avoided in patients with liver disease.
6. DV should be avoided in patients with renal disease.
7. DV separates from placebo by week 1 in menopausal women with hot flushes.
8. The 100 mg/day dose of DV optimizes safety and adverse effects in the treatment of the vasomotor symptoms of menopause.

## CME ANSWERS

1. False; 2. True; 3. False; 4. True; 5. False; 6. False; 7. True; 8. True.

1. DV blocks the synaptic reuptake of serotonin and norepinephrine. As norepinephrine and acetylcholine have opposing actions, increased noradrenergic activity simulates anticholinergic activity and results in effects such as dry mouth and constipation. Such effects are seen with other noradrenergic antidepressant as well, including reboxetine, milnacipran and duloxetine. DV does not block muscarinic receptors. In fact, with the exception of its inhibition of the serotonin and norepinephrine transporters, DV has negligible effects on 96 different transporters, receptors, enzymes, ion channels, second messengers, and other molecular targets.[13]
2. In randomized controlled trials, treatment with DV was dose-dependently associated with weight loss of around 0.5-1.0 kg across an 8-week treatment period.[9]
3. In a pooled analysis of nine clinical trials, DV was shown to slightly but (statistically) significantly increase the QTc interval; however, the magnitude of increase was small, clinically insignificant and could at least partly be explained by the application of the Bazett formula for QT correction. The Bazett formula overcorrects the QT interval when tachycardia is present, as was the case in the DV clinical trials.[9] In an as-yet unpublished study of healthy female volunteers, DV did not prolong the Fridericia-corrected QT interval.[1]
4. DV inhibits CYP 2D6, but the magnitude of inhibition is unlikely to be of clinical significance in most patients. In comparisons between DV and duloxetine[14] or paroxetine,[15] it was observed that the *in vivo* inhibition of CYP 2D6 was slight with DV (100 mg/day), modest with duloxetine (60 mg/day) and pronounced with paroxetine (20 mg/day). At doses of 400 mg/day, however, DV may be associated with a greater degree of CYP 2D6 inhibition.[16] In the approximately 10% of subjects who are CYP 2D6 poor metabolizers, such inhibition could be clinically significant. Of note, CYP 2D6 is responsible for the metabolism of tricyclic antidepressant (TCA), beta-blockers, many antipsychotic drugs, opiates, anti-arrhythmic agents, tamoxifen and, in general, about 20-25% of the pharmacopoeia.
5. DV is only about 30% protein-bound.[7] Therefore, a decrease in plasma protein levels associated with liver disease is unlikely to much influence the free:bound fraction of DV. Less than 5% of an administered dose of DV is metabolized in the liver; the enzyme involved is CYP 3A4 and the metabolite is N, O-didesmethylvenlafaxine.[7] About 20% of DV is conjugated by glucuronidation,[7] a pathway that is affected only in more severe liver disease. Thus, the liver plays a relatively minor role in DV pharmacokinetics. The drug could therefore be expected to be safe in liver disease. Indeed, the half-life and area under the curve (AUC) are increased by only about 30-40% in patients with moderate to severe liver disease.[17] The Wyeth prescribing information for DV therefore indicates that no dose adjustments are necessary; however, caution is always advisable. In this context, several points are worthy of note:
  - In the DV clinical trials, doses of up to 400 mg/day were studied for durations of up to 8 weeks.[1,7,9] These doses would result in considerably higher blood levels than those associated with DV doses of 50-100 mg/day in the presence of liver disease. As the 200-400 mg/day doses were reasonably well tolerated by most patients,[7] it is likely that the 50-100 mg/day doses of the drug would be safe and well tolerated even when liver functioning is impaired.
  - In the DV clinical trials, small but statistically significant increases in alkaline phosphatase and transaminase enzymes levels were observed across an 8-week treatment period.[9] Reassuringly, as yet unpublished data indicate that these increases do not magnify during long-term treatment and that the elevated enzyme levels return to baseline after discontinuation of DV.[9] There is a theoretical risk that this effect on liver enzymes could assume clinical significance in patients whose liver function has already been compromised by other insults.
  - Reassuringly, DV does not inhibit the effects of CYP 1A2, CYP 2A6, CYP 2C8, CYP 2C9, CYP 2C19, and CYP 3A4;[18] i.e., enzymes responsible for the metabolism of about three-quarters of the pharmacopoeia. However, as already mentioned, clinical (50-100 mg/day) doses of DV will result in a small degree of inhibition of CYP 2D6.[14,15] This inhibition is too small to be of clinical importance in persons with a healthy liver; however, it could result in the potential for CYP 2D6 drug interactions in those whose liver is already compromised by other insults. The potential for drug interactions could be even greater when the dose of DV is higher, such as 400 mg/day.[16]
6. The Wyeth prescribing information indicates that, in mild to moderate renal disease, the DV AUC is increased by 42-46%; in severe and end-stage renal disease, the AUC is increased by 108-116% and when creatinine clearance is <30 ml/min, clearance of DV is reduced by half. Thus, the pharmacokinetics of DV in severe and end-stage renal disease are akin to a doubled dose of the drug. Considering that doses of up to 400 mg/day (i.e., up to eight times the recommended dose of 50 mg/day) have been studied and found safe in 8-week clinical trials, doubled DV levels may not be of much concern. Therefore, whereas no dose adjustments are necessary when renal disease is mild to moderate, when renal disease is severe, halving the daily dose or shifting to alternate-day dosing at the same dosage level would suffice.[1]
7. DV has an early onset of action against the vasomotor symptoms of menopause; a statistically significant advantage over placebo is apparent by the end of week 1 itself.[3–5] This response is maintained at 12 weeks[3–5] and 26 weeks.[5] The number needed to treat for 75% reduction in moderate to severe hot flushes is approximately 5.
8. Speroff *et al*.[3] found that DV separated from placebo in doses of 100,150 and 200 mg/day but not 50 mg/day. The 100 mg/day dose optimized the balance between efficacy and adverse effects. Two other studies[4,5] also reported 12-week outcomes with statistically indistinguishable improvement at doses of 100 and 150 mg/day. However, one study[5] found that only the 150 mg/day dose remained superior to placebo at 26 weeks, but this study had only been powered for a 12-week treatment endpoint. It may be noted that DV has not yet been approved for the treatment of menopausal symptoms.
